# Combining Electrostimulation With Fiber Tracking to Stratify the Inferior Fronto-Occipital Fasciculus

**DOI:** 10.3389/fnins.2021.683348

**Published:** 2021-05-20

**Authors:** Alexandre Roux, Anne-Laure Lemaitre, Jeremy Deverdun, Sam Ng, Hugues Duffau, Guillaume Herbet

**Affiliations:** ^1^Department of Neurosurgery, GHU Paris, Sainte-Anne Hospital, Paris, France; ^2^Université de Paris, Sorbonne Paris Cité, Paris, France; ^3^Inserm UMR 1266, IMA-Brain, Institute of Psychiatry and Neurosciences of Paris, Paris, France; ^4^Department of Neurosurgery, Gui de Chauliac Hospital, Montpellier University Medical Center, Montpellier, France; ^5^Department of Neuroradiology, Gui de Chauliac Hospital, Montpellier University Medical Center, Montpellier, France; ^6^I2FH, Institut d’Imagerie Fonctionnelle Humaine, Gui de Chauliac Hospital, Montpellier University Medical Center, Montpellier, France; ^7^Institute of Functional Genomics, University of Montpellier, INSERM, CNRS, Montpellier, France

**Keywords:** diffuse glioma, non-verbal semantic cognition, mentalizing, inferior fronto-occipital fasciculus, electrostimulation

## Abstract

The inferior fronto-occipital fasciculus (IFOF) is one of the longest association fiber tracts of the brain. According to the most recent anatomical studies, it may be formed by several layers, suggesting a role in multiple cognitive functions. However, to date, no attempt has been made to dissociate the functional contribution of the IFOF subpathways. In this study, real-time, cortico-subcortical mapping with direct electrostimulation was performed in 111 patients operated on in wide-awake surgery for a right low-grade glioma. Patients performed two behavioral tasks during stimulation, tapping, respectively, mentalizing and visual semantic cognition—two functions supposed to be partly mediated by the IFOF. Responsive white matter sites were first subjected to a clustering analysis to assess potential topological differences in network organization. Then they were used as seeds to generate streamline tractograms based on the HC1021 diffusion dataset (template-based approach). The tractograms obtained for each function were overlapped and contrasted to determine whether some fiber pathways were more frequently involved in one or the other function. The obtained results not only provided strong evidence for a role of the right IFOF in both functions, but also revealed that the tract is dissociable into two functional strata according to a ventral (semantic) and dorsal (mentalizing) compartmentalization. Besides, they showed a high degree of anatomo-functionnal variability across patients in the functional implication of the IFOF, possibly related to symmetrical/hemispheric differences in network organization. Collectively, these findings support the view that the right IFOF is a functionally multi-layered structure, with nevertheless interindividual variations.

## Introduction

The inferior fronto-occipital fasciculus (IFOF) is one of the longest association fiber tract of the brain; it interconnects the frontal lobe to different territories of the posterior cortex (Schmahmann and Pandya, 2007). Although the earliest descriptions of the IFOF are long-lasting (Burdach, 1822; Dejerine and Dejerine-Klumpke, 1895; Trolard, 1906; Curran, 1909), the interest for this ventral fasciculus has renewed only recently—especially because of the revival of post-mortem anatomical dissection techniques (Martino et al., 2010; Sarubbo et al., 2013) and the development of tractographic imaging that allows indirect visualization of fiber pathways (Catani et al., 2002; Mori et al., 2002, 2005; Schmahmann and Pandya, 2007; Thiebaut de Schotten et al., 2012; Caverzasi et al., 2014). The IFOF has been thought to be specific to humans, but recent evidence indicates that it may be in fact present in the monkey brain (Sarubbo et al., 2019).

Anatomical studies suggest that the IFOF is not a monolithic white matter (WM) tract, but rather organized into distinct anatomical strata. The first modern dissection work has shown that the IFOF may be split into two layers, a superficial one and a deeper one (Martino et al., 2010). The former connects the frontal lobe to both the superior parietal lobule and the postero-superior part of the occipital lobe, whereas the latter interconnects the frontal lobe with the postero-inferior part of the occipital lobe and the postero-basal temporal areas. More recently, a study combining anatomical dissection and diffusion imaging has allowed disentangling the frontal projections of the two layers (Sarubbo et al., 2013). The superficial layer terminates in the inferior frontal gyrus, while the deep one ends in different prefrontal areas thus forming different subpathways: the anterior sublayer has cortical terminations in both the orbito-frontal cortex and the frontal pole, the middle sublayer in the lateral orbito-frontal cortex and the middle frontal gyrus, and the posterior sublayer mainly in the dorsolateral prefrontal cortex. According to another work using diffusion spectrum imaging, the connective architecture of the IFOF may be even more complex than previously thought, with possibly five layers that would be “trackable” from their prefrontal terminations (Wu et al., 2016). Other studies confirmed that the IFOF projects into many areas of the brain; among them, some were not previously identified as receiving connective inputs from the tract such as the angular gyrus or the posterior temporal cortex (Caverzasi et al., 2014; Hau et al., 2016; Wu et al., 2016). These studies also showed that the IFOF cortical terminations are highly variable across individuals, with the exception of projections targeting the inferior frontal, lateral orbito-frontal, and lateral occipital gyri (Hau et al., 2016).

This multi-layered organization agrees with the array of functions that have been assigned to the IFOF. Neuromodulation studies using direct electrostimulation mapping, the only technique allowing direct access to tract functions (Duffau, 2015), have demonstrated a role of this connection in verbal and visual semantic cognition (Duffau et al., 2005; Moritz-Gasser et al., 2013; Herbet et al., 2017a), and mentalizing (Yordanova et al., 2017), a finding that have been now replicated in lesion mapping works (Almairac et al., 2015; Mirman et al., 2015; Marino Dávolos et al., 2020). Other studies suggested a role in emotion recognition (Philippi et al., 2009; Crespi et al., 2014), spatial cognition and neglect (Urbanski et al., 2008; Herbet et al., 2017b), face recognition (Thomas et al., 2009) and orthographic processes (Vandermosten et al., 2012; Wang et al., 2020). However, neither of these studies has attempted and succeeded to relate the putative functions of the IFOF to specific sublayers.

With the purpose of investigating whether distinct right IFOF sublayers might be specifically devoted to either mentalizing or visual semantic cognition, we analyzed a unique dataset gained from direct brain stimulation mapping achieved in awake patients, combined with q-space diffusion-based fiber-track imaging. The secondary objective was to explore the existence of potential interindividual variations in the functional compartmentalization of the IFOF.

## Materials and Methods

### Patients

All patients recruited in this study were consecutively operated on for a histopathologically proven supratentorial diffuse low-grade glioma at Montpellier University Hospital’s Department of Neurosurgery over a period of 5 years. They were included if they met the following criteria: (i) a surgical resection performed in “awake” condition with a cortico-sub-cortical mapping by means of direct electrostimulation; (ii) a glioma close to or infiltrating at least in part the IFOF in the right hemisphere; (iii) an intraoperative mapping of mentalizing and/or visual semantic cognition (see below for a comprehensive description of the behavioral tasks); and (iv) available 3-month postoperative anatomical MRI. Patients showing a preoperative impairment of mentalizing or visual semantic cognition not allowing to objectively probe these functions during the mapping were excluded (no patients were excluded by this criterion).

A total of 189 patients with a right supratentorial low-grade diffuse glioma were initially screened. Among those, we excluded 71 patients for whom the IFOF was not stimulated during the surgical procedure because the tumors were distant from the tract and its interconnected areas. The final sample thus consisted of 111 patients (46 females and 65 males), with a mean ± SD age of 39.8 ± 10.4 years (range: 23–70 years). All patients except 18 were right-handed (5 ambidextrous and 13 left-handed).

### “Awake” Surgery Procedure

All patients included in this study benefited from a “wide-awake” surgical procedure performed by the same well-experienced neurosurgeon (HD). An intraoperative multifunctional mapping was systematically achieved at both the cortical and axonal levels by means of direct electrostimulation. The general principles of such a surgical approach has been comprehensively described by our research group in another works (Duffau et al., 2005; Tate et al., 2014). Briefly, after craniotomy and dural opening under general anaesthesia, the patient was awoken and the cortical surface was exposed. Next, the tumor gross limits were identified by intraoperative ultrasound and visually indicated with sterile letter tags, placed directly on the cortex. Direct electrical stimulations were then performed using a biphasic electric current (60 Hz frequency, 1 ms pulse-width, amplitude 2–5 mA) delivered by a bipolar probe with 5 mm inter-tip space (NIMBUS Stimulator, Newmedic, France). A sensorimotor and speech mapping was first performed. A small current was initially used and incrementally increased up by step of 0.5 mA until a reliable speech arrest, or motor or sensory response was triggered. This stimulation threshold was maintained during the remaining of the cortical and subcortical mapping. The cerebral cortex was then mapped with different behavioral tasks, including tasks tapping into aspects of mentalizing and visual semantic cognition. To both limit the spread of current through the brain tissue and preserve response specificity, the length of stimulation never exceeded 4 s. All the exposed cortical surface was mapped, and cortical areas whose stimulations induced three non-consecutive, identical perturbations was accepted as responsive for the function under scrutiny (Ojemann and Mateer, 1979); they were subsequently marked with sterile tags. Patients’ functions were monitored by a senior clinical neuropsychologist, blinded to when stimulations were applied. After the completion of cortical mapping, several photographs were taken for off-line treatment of data. Then, glioma removal was started. With the progress of resection, the WM tracts were gradually accessible for stimulation. WM sites associated with recurrent functional responses were marked with sterile tags. New photographs from different angles of view were taken.

### Intraoperative Behavioral Paradigms

**FIGURE 1:**
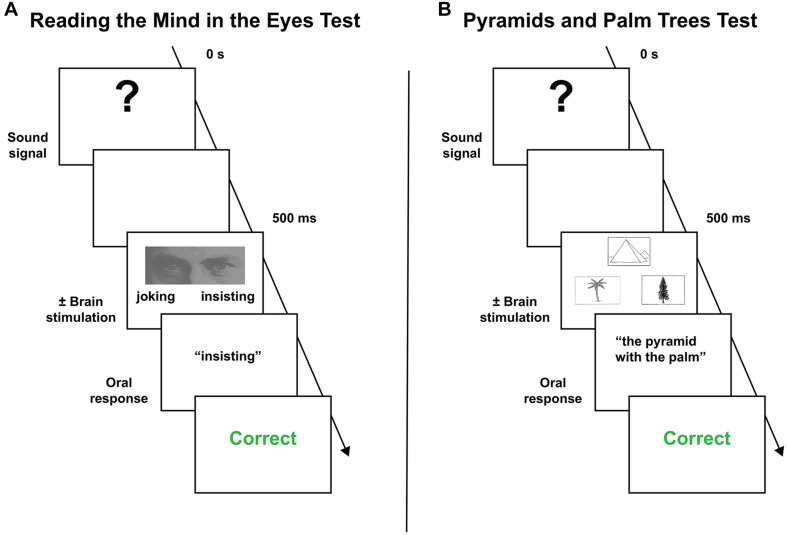
Behavior paradigms employed during the intraoperative mapping. **(A)** Mentalizing task. **(B)** Visual semantic cognition task (see the main text to a complete description of the behavioral procedure).

The behavioral paradigm to assess mentalizing “on-line” has been previously described in another study (Herbet et al., 2015) and is schematically illustrated in Figure 1A. It is an adapted version of the well-used “Read the Mind in the Eyes” task (hereafter, RME), initially developed by Baron-Cohen et al. (2001) to gauge social intelligence in autistic patients. In the standard version of the task, the participant is asked to choose among four complex mental states the one that best matches with what the person seen on a photograph is feeling or thinking. Only the eye region of the face is shown. This version, composed of 36 items, was systematically achieved the day before surgery. Due to the inherent complexity of the RME task, only two mental states were used during the intraoperative mapping in order to decrease the number of false positive manifestations and thus the number of required stimulations. For the same reason, only succeeded items during the preoperative assessment were included in the intraoperative protocol.

To assess visual semantic cognition, the Pyramids and Palm Trees test (hereafter, PPTT) was used (Howard et al., 1992). This force-choice task, in which the participant is required to form a semantic association, comprises 52 black and white items in its standard version. For each target item, two further pictures are proposed. The patient is asked to select the one that best matches with the target picture according to the most straightforward semantic link. As for the RME task, the PPTT was achieved the day before surgery, and impaired patients were dismissed from the mapping. Items associated with incorrect responses were excluded from the intraoperative materials. The intraoperative protocol is described in Figure 1B.

To demonstrate specificity, and depending on which networks were damaged by the tumor, anatomical sites associated with mentalizing and/or visual semantic impairments were also stimulated while the patients were performing other behavior tasks. This included a naming task (Duffau et al., 2005), a line bisection task (Herbet et al., 2017b), a motor task (Rech et al., 2019), and a passive somatosensory task (Tate et al., 2014). In this study, only semantic and mentalizing sites are reported.

It is worth mentioning that, on the total of patients included (*n* = 111), 104 (93.7%) patients were intraoperatively assessed for visual semantic cognition, 90 (81.1%) for mentalizing and 83 (74.8%) for both functions.

### Imaging Acquisition and Spatial Normalization

Structural MRI datasets were collected 3 months after surgery on either a 1.5T (Avanto, Siemens Medical Systems, Erlangen, Germany) or a 3T MR imaging scanner (Skyra, Siemens Medical Systems, Erlangen, Germany), as part of the routine management protocol. For the purpose of the current work, standard 3D gadolinium-enhanced axial T1-weighted images were used. The 1.5T/3T MRI parameters were as follows: repetition time 1,880/1,700 ms; echo time 3.4/2.5 ms; inversion time 1,100/922 ms; field of view 256 × 256/250 × 250 mm; flip angle 15/9°; voxel size 1 × 1 × 1 mm^3^ and; 176 axial slices.

All individual MRI datasets were registered to the Montreal Neurological Institute (MNI) 152 template with an isotropic resolution of 1-mm. This work was achieved using SPM12 software^1^ implemented in MATLAB environment (Matrix Laboratory, MathWorks, Inc.—R2018a), with standard parameters. At this stage, all MRIs were visually inspected to ensure sufficient quality. All registrations were satisfactory.

### Distribution of Stimulation-Related Anatomical Sites

The coordinates of each anatomical site was manually plotted onto the patients’ respective normalized MRI. They were established by using a combination of intraoperative photos taken from different points of view (allowing to precisely identify the location of functional sites indicated by sterile numbered tags), written surgical reports, cortical (e.g., gyri, sulci and vessels) and subcortical (e.g., edges of the resection cavity) landmarks identifiable on both the normalized MRI and the intraoperative photos. This method has been successfully applied in recent studies (e.g., Sarubbo et al., 2020). The accuracy of coordinate positioning was systematically checked by the senior author. When a disagreement occurred, the location of problematic sites was remapped in a consensual manner. The MNI coordinates (x, y, z) were recorded in separate databases as a function of the behavior tasks (i.e., PPTT or RME or both) and the stimulation location (i.e., cortical vs. subcortical). Then, for each stimulation site, a spherical volume of interest (VOI) was generated using the MarsBaR toolbox^2^ with a radius of 2.5 mm corresponding to the spatial resolution of the bipolar stimulator.

To demonstrate reliability in the way stimulation sites were positioned, we asked an independent operator with certified expertise in neurosurgery and neuroanatomy, and fully blind to both the general purpose and the results of the study, to position again 40 subcortical sites randomly selected among the 105 subcortical sites already positioned by the first operator (i.e., subcortical sites either associated with mentalizing or visual semantic cognition during electrostimulation). To obtain a quantitative index of inter-operator reliability, three parametric correlations were performed, one for each axis. We further statistically compared the coordinates between both operators. We also calculated both the mean and the standard deviation of the absolute difference in site estimates (one for each axis) in order to provide a measure of error (see Herbet et al., 2019 for a comparable method).

### Clustering Analysis of Subcortical Data

In view of the apparent topological heterogeneity between the distribution of the mentalizing and the semantic subcortical sites, at least in the WM deep in the prefrontal and the posterior parietal cortices (see below), a clustering analysis was conducted with each stimulation dataset. As a first step, we used the Duda-Hart procedure (Duda and Hart, 1973) to decide whether the data should be split into two or more clusters. Then, when the null hypothesis of homogeneity was rejected at *p* < 0.05, we performed a formal clustering analysis with the *k-*means algorithm, by varying *k* from 2 to 10 with 100 iterations. Clusters were formed by observations that maximize between-cluster distances using a squared Euclidean distance-based measure. The optimal number of clusters was estimated with a leave-one-out cross-validation procedure. To assess the statistical differences between the clusters in each stimulation dataset, an ANOVA was performed for each coordinate. The analyses were mainly achieved with SPSS^3^ and the “Flexible Procedures for Clustering” (FPC) package (Hennig, 2020) implemented in *R* environment^4^.

### Disconnection Analysis: A Population-Based Approach With the HCP-1021 Diffusion Data

Our aim here was to dissociate the WM fiber connections specific either to mentalizing or visual semantic cognition. As a first step, a population-based template was constructed based on the diffusion data of 1,021 participants from the Human Connectome Project (2017 Q4, 1,200-subject release). A multishell diffusion scheme was used; the *b*-values were 1,000, 2,000, and 3,000 s/mm^2^. The number of diffusion-sampling directions was 90 for each *b*-value. The in-plane resolution, as well as the slice thickness, was 1.25 mm. The diffusion data were reconstructed in the MNI space using *q*-space diffeomorphic reconstruction (Yeh and Tseng, 2011) to obtain the spin distribution function (Yeh et al., 2010). A diffusion sampling length ratio of 2.5 was used, and the output resolution was 2 mm. The restricted diffusion was quantified using restricted diffusion imaging (Yeh et al., 2017). The analysis was conducted using DSI Studio^5^.

Each 5-mm (in diameter) subcortical spherical VOI was used as seeds for fiber tracking allowing us to generate a map of the fiber connections passing through or originating from this VOI. The same tracking parameters were maintained across all tractographies performed; this included a tracking threshold automatically determined using the Otsu’s method, an angular threshold of 55°, a step size of 1 mm, a minimal and maximal length of 10 and 300 mm, respectively. To estimate fiber direction, the standard trilinear interpolation method was selected. Streamlines were tracked using the Euler Algorithm.

Once all tractographies were performed, they were transformed into binarized maps. These maps were overlaid according to the associated impairment (i.e., mentalizing *or* visual semantic cognition) using MRIcron software. Then MRIcron’s subtraction plot function was employed to identify the WM voxels the most frequently and specifically “disconnected” during mentalizing vs. semantic electrostimulation, and *vice versa*.

### Standard Protocol Approvals, Registrations and Patient Consents

Data were acquired as part of the current clinical practice and were retrospectively studied. Patients consented to the extraction of data from their medical records. We conducted this study in compliance with the ethical standards of our institution for a retrospective study.

## Results

### Patients’ Clinical Characteristics and Tumor Topography

A total of 111 patients (46 females) presenting with a right supratentorial diffuse low-grade glioma fulfilled all inclusion criteria and thus constituted the final sample. An overview of the patients’ sociodemographic and clinical data is provided in Table 1. Consistent with the preferential distribution of low-grade glioma (Herbet et al., 2016), the most common location was in the frontal and the insular cortex. The mean pre-operative tumor volume was 51.0 ± 41.7 cm^3^.

**TABLE 1:** Main characteristics of the study sample.

**Parameters**	***n***	**%**
**Clinical characteristics at diagnosis**		
**Sex**		
Female	46	41.4
Male	65	58.6
**Age, years (mean, SD) 39.8 ± 10.4**		
≤40	64	57.7
>40	47	42.3
**Handedness**		
Right-handed	93	83.8
Left-handed	13	11.7
Ambidextrous	5	4.5
**Presenting symptom**		
Incidental	29	26.1
Epileptic seizure	80	72.1
Neurological focal deficit	2	1.8
**Imaging characteristics at diagnosis**		
**Topography of tumor**		
Frontal	45	40.6
Fronto-temporo-insular	25	22.5
Temporal	12	10.8
Parietal	7	6.3
Fronto-insular	6	5.4
Temporo-insular	4	3.6
Temporo-occipital	3	2.7
Insular	3	2.7
Fronto-cingular	3	2.7
Fronto-temporo-parieto-insular	2	1.8
Fronto-parieto-insular	1	0.9
**Tumor volume, cm^3^ (mean, SD) 51.0 ± 41.7**		
<50	67	60.4
≥50	44	39.6
**Treatment-related characteristics**		
** Extent of resection, percent (mean, SD) 92.8 ± 8.8**		
<90%	29	26.1
≥90%	82	73.9

### Inter-Operator Reliability in Site Estimates

The results from correlation analyses performed between the MNI coordinates of each operator indicated *r*_40_ = 0.93, *r*_40_ = 0.99 and *r*_40_ = 0.97 for, respectively, the *x*-, *y*-, and *z*-axis. No significant difference was found in estimates between both operators for the *x*- (*t*_39_ = 0.14, *p* = 0.89), *y*- (*t*_39_ = 1.57, *p* = 0.12) and *z*-axis (*t*_39_ = 0.90, *p* = 0.37). The mean ± SD of the absolute difference between both operators was 0.08 mm ± 3.36 for the *x*-axis, 1.27 mm ± 5.12 for the *y*-axis, and 0.87 mm ± 6.1 for the *z*-axis. These analyses clearly indicate that the positioning work was reliable and accurate.

### Topological Organization of Cortical Sites

**FIGURE 2:**
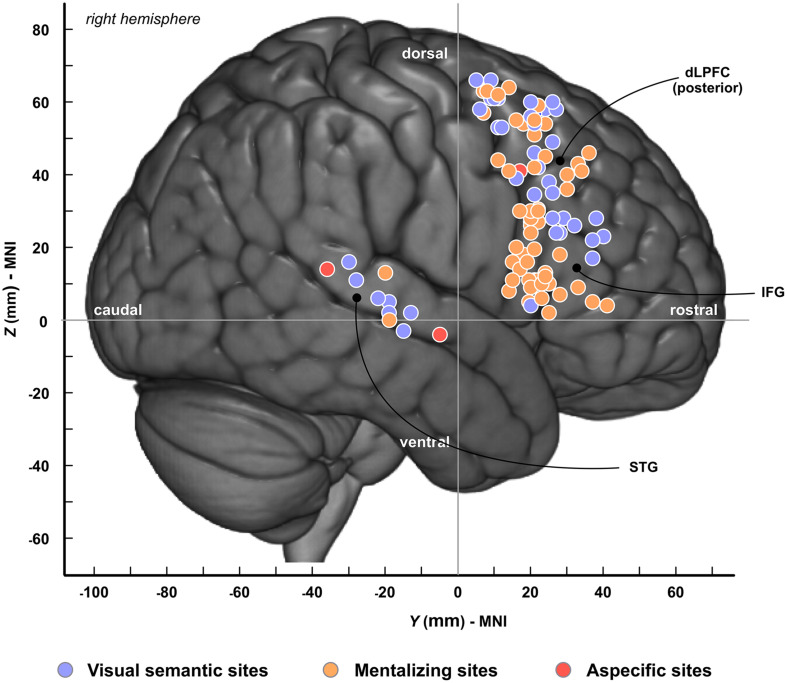
Spatial topography of cortical sites. Cortical sites are plotted onto the MNI-152 template. Colors refer to the type of neuropsychological impairments induced by electrostimulation. Blue, visual semantic cognition; Orange, mentalizing; Red, sites associated with both visual semantic cognition and mentalizing. dlPFC, dorsolateral prefrontal cortex; IFG, inferior frontal gyrus; STG, superior temporal gyrus.

In total, 101 cortical sites were identified across patients; among these, 57 were specifically associated with mentalizing (Figure 2, orange circles), 41 with visual semantic cognition (Figure 2, blue circles) and 3 with both functions (called “aspecific sites”) (Figure 2, red circles). Mentalizing sites were distributed in IFG (pars opercularis, *n* = 24; pars triangularis, *n* = 10; pars orbitalis, *n* = 1), in dlPFC (*n* = 20) and in the superior temporal gyrus (STG, *n* = 2). Semantic sites were mainly located in dlPFC (*n* = 30), and to a lesser extent in the mid-to-posterior STG (*n* = 7) and in IFG (pars opercularis, *n* = 1; pars triangularis, *n* = 3). Among the three “aspecific” sites, two were located in STG and one in dlPFC. To summarize, the spatial distribution of mentalizing and semantic sites were relatively similar across patients, except for IFG that was clearly under-represented for visual semantic cognition.

### Topological Organization of Subcortical Sites

**FIGURE 3:**
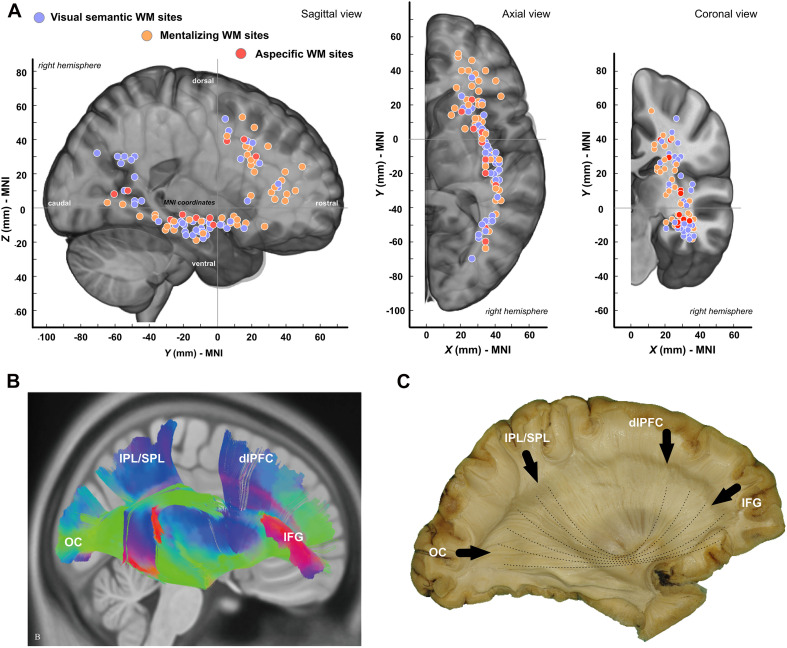
Spatial distribution of white matter sites. **(A)** Tridimensional distribution of WM sites. **(B)** Template-based fiber tracking of the right IFOF with q-diffusion imaging (from Wu et al., 2016). **(C)** Anatomical dissection of the right IFOF (personal materials). OC, occipital pole; IPL, inferior parietal lobule; SPL, superior parietal lobule; dlPFC, dorsolateral prefrontal Cortex; IFG, inferior frontal gyrus.

Overall, 105 sites were detected during subcortical stimulation: 46 were specific to mentalizing, 49 to semantic cognition, and 10 were associated with both functions. The distribution of these anatomical sites is provided in Figure 3A, the vast majority of which were located along the topological positioning of the IFOF according to the most recent anatomical studies using either DTI (Figure 3B) or post-mortem dissection (Figure 3C). Visually, although the two distributions were quite similar, though, two differences were noticeable: indeed, mentalizing sites were over-represented in the WM fibers underlying the anterior part of both dlPFC and pars triangularis whereas visual semantic cognition were rather over-represented in the WM fibers of the posterior parietal cortex (Figure 3A).

### Relationships Between Cortical and Subcortical Sites, and Between Functions

In total, 83 patients benefited from a mapping concerning both mentalizing and visual semantic cognition. At the cortical level, at least one anatomical site for each function was identified in 16 patients (19.3%). Conversely, 14 (16.9%) patients uniquely showed one (or more) site(s) for visual semantic cognition, and 35 (42.2%) for mentalizing. Note that no cortical responses were found in 18 patients (21.6%) for these functions.

As regards with subcortical mapping, 16 patients (19.3%) showed at least one anatomical site for each function. 16 (19.3%) patients uniquely showed one (or more) site(s) for visual semantic cognition, and 33 (39.8%) for mentalizing. No subcortical responses were found in 18 patients (21.6%).

Collectively, these results show a high inter-individual variability in the functional involvement of the IFOF with, nevertheless, a more frequent implication for mentalizing vs. visual semantics (2-tailed *t*-test for proportion comparison, *p* = 0.004). Interestingly, this variability echoed those identified at the cortical level with, again, an over-involvement of mentalizing vs. visual semantic cognition (*p* = 0.002).

As a significant proportion of patients was left-handed, we checked whether the distribution of functional sites could be considered as equivalent between left-handed and right-handed patients. The proportion of unmasked cortical sites were not different for mentalizing (*p* = 0.46) and visual semantic cognition (*p* = 0.78). However, although no difference was revealed for semantic cognition at the subcortical level (*p* = 0.21), this was not the case for mentalizing (*p* = 0.017) (left-handed patients showed a lower rate of subcortical sites). This suggests that the right white matter network is less engaged in left-handed patients for mentalizing.

### Clustering Analysis Results

**FIGURE 4:**
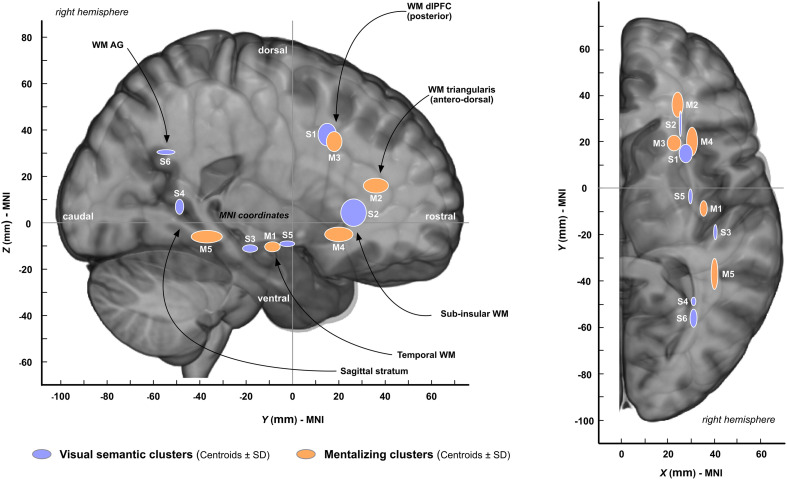
Results from the clustering analysis. The coordinates of each cluster are represented by centroids ± SD (see Table 2 for the precise MNI coordinates). “S” means “semantic cluster” and “M” means “mentalizing cluster.” AG, angular gyrus; dlPFC, dorsolateral prefrontal cortex.

Both mentalizing and non-verbal semantic cognition stimulation datasets were allowed to be clustered according the Duda-Hart statistics (*p* < 0.05). For mentalizing, a 5-cluster solution model was found to best fit the data (training error = 0.037). The MNI coordinates between the five clusters were significantly different (*x*: *F* = 559.01, *p* < 0.001; *y*: *F* = 77.48, *p* < 0.001; *z*: *F* = 104.16, *p* < 0.001). For visual semantic cognition, a 6-cluster solution model was estimated as the most appropriate for the data (training error = 0.033) and, again, the between-coordinates differences were significant (*x*: *F* = 326.28, *p* < 0.001; *y*: *F* = 47.18, *p* < 0.001; *z*: *F* = 99.68, *p* < 0.001). The centroids ± SD of the clusters are reported in Table 2 and plotted in Figure 4. To summarize, a high topological similarity was found for both functions in the prefrontal dorsolateral, subinsular/anterior extreme capsule and temporal WM. Dissimilarities were, however, observed in the WM of the antero-dorsal pars triangularis (cluster specific to mentalizing) and in WM of the angular gyrus (cluster specific to visual semantic cognition).

**TABLE 2:** Cluster coordinates.

**Parameters**	**Clusters**	**Clusters location**	**Number of cases for clustering**	**MNI coordinates ± SD**		
				***X***	***Y***	***Z***
Mentalizing	Cluster 1	Temporal	8	35 ± 3	−9 ± 7	−11 ± 4
	Cluster 2	Antero-dorsal pars triangularis	9	24 ± 5	37 ± 11	16 ± 6
	Cluster 3	Posterior dlPFC	11	23 ± 6	18 ± 7	36 ± 9
	Cluster 4	Subinsular/anterior extreme capsule	8	31 ± 5	20 ± 12	−5 ± 6
	Cluster 5	Sagittal stratum	10	40 ± 3	−37 ± 13	−6 ± 5
Visual semantic cognition	Cluster 1	Posterior dlPFC	6	28 ± 6	15 ± 8	38 ± 10
	Cluster 2	Subinsular/anterior extreme capsule	4	26 ± 1	27 ± 11	5 ± 12
	Cluster 3	Temporal	13	41 ± 2	−19 ± 7	−12 ± 4
	Cluster 4	Sagittal stratum	5	36 ± 2	−49 ± 4	8 ± 7
	Cluster 5	Temporal	15	34 ± 2	−3 ± 6	−11 ± 2
	Cluster 6	Angular gyrus	6	31 ± 3	−56 ± 8	29 ± 2

*SD, standard deviation; dlPFC, Dorsolateral Prefrontal Cortex; MNI, Montréal Neurological Institute.*

### Disconnection Analysis

**FIGURE 5:**
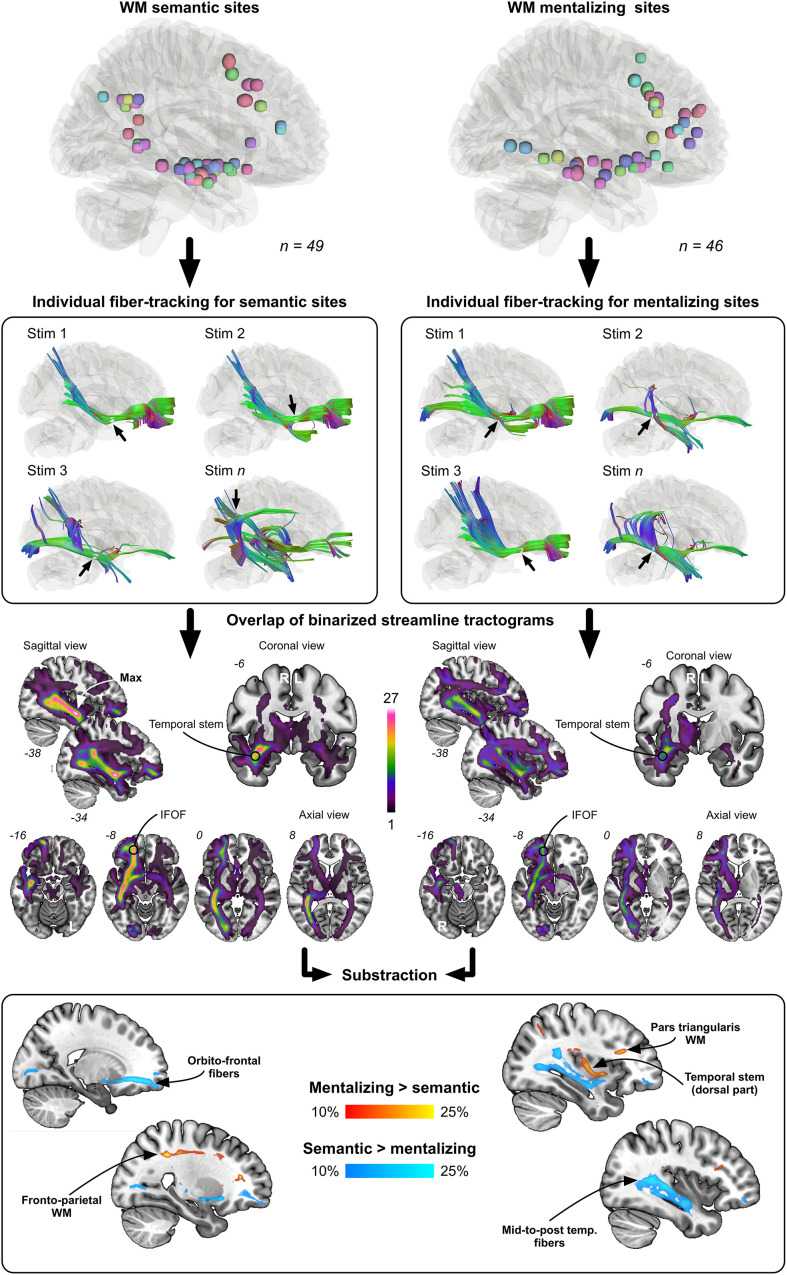
Results from the disconnection analysis. Each WM site was used to generate individual streamline tractograms. These tractograms were next binarized and overlapped according to each function (semantics vs. mentalizing). Last, the overlap maps were contrasted to visualize the fiber pathways the most frequently and specifically tracked from mentalizing sites (red to yellow color) and semantic sites (dark blue to light blue color).

The disconnection analysis, summarized in Figure 5, first confirmed that the most “disconnected” voxels during stimulations associated with either mentalizing or visual semantic cognition mainly belonged to the right IFOF. However, the subtraction between mentalizing and visual semantic cognition binarized streamline tractograms (and vice versa) revealed some noticeable differences: WM semantic sites were more frequently crossed by orbitofrontal and mid-to-post temporal IFOF streamlines whereas WM mentalizing sites were more frequently crossed by fronto-parietal and dorsal temporal stem/extreme/external capsule streamlines. These differences were moderate because not exceeding 25%.

## Discussion

By virtue of their brain-wide cortical projections, association fiber pathways are vital building blocks in integrating information within and across neurocognitive networks (Herbet and Duffau, 2020). Until recently, association tracts were frequently viewed as monolithic structures, the primary consequence of which was a tendency to conceptualize them as monofunctional pieces. However, recent works using tractography imaging or anatomical dissection has provided evidence that association tracts are in fact formed by multiple anatomical layers and thus are possibly involved in different functions (Martino et al., 2010; Wu et al., 2016; Latini et al., 2017; Herbet et al., 2018). The IFOF is no exception to the rule, as its connective architecture is complex. This tract may indeed be segregated into two (Martino et al., 2010; Sarubbo et al., 2013) or more (Wu et al., 2016) sublayers with distinct frontal or posterior terminations. In this context, the present study was an attempt to dissociate two functional pathways into the right IFOF with the major advantage of using stimulation mapping—the sole available method allowing direct functional interrogation of WM tracts (Duffau, 2015).

First of all, the results confirmed that the right IFOF is a critical WM connection in the networks supporting, respectively, visual semantic cognition and mentalizing, as suggested in other neuromodulation (Moritz-Gasser et al., 2013; Herbet et al., 2017a; Yordanova et al., 2017) or neuroimaging studies (Mirman et al., 2015; Grosse Wiesmann et al., 2017). Cortical sites were indeed observed in structures known to receive connective inputs from the IFOF, including posterior dlPFC, IFG and STG (Sarubbo et al., 2013; Caverzasi et al., 2014; Hau et al., 2016; Wu et al., 2016). Most importantly, the subcortical sites were mainly situated along the typical course of the IFOF (Figure 3). Furthermore, the overlap maps of individual tractograms showed that streamlines generated from subcortical sites belonged in average to the right IFOF, again, for both functions. As matter of fact, the maximum overlaps occurred at the level of the temporal stem, a classical anatomical reference not only to identify the tract during neurosurgery (Duffau et al., 2005) but also to track it with diffusion imaging (steam-based approach)—as the entire range of IFOF fibers pass through this region (Hau et al., 2016; Wu et al., 2016).

The main goal of this study was to determine whether the IFOF might be dissociated into (at least) two anatomical strata, each of them specifically devoted either to semantic cognition or to mentalizing. The results obtained support at least partially this hypothesis. First, consistent with the general distribution of subcortical sites (Figure 3), subcortical clustering analyses revealed that mentalizing sites were over-represented deep in the white matter underlying pars triangularis whereas semantic sites were rather over-represented in the depth of the angular gyrus, suggesting that the IFOF fibers involved in each function are dissociable, but only at the level of the terminal parts of the tract (Figure 4). Second, the subtraction analyses indicated (i) that semantic sites were more frequently crossed by ventral streamlines compared to mentalizing sites, including IFOF orbito-frontal and mid-to-posterior temporal fibers; (ii) conversely that mentalizing sites were more frequently crossed by more dorsal streamlines compared to semantic sites, including fibers passing through the dorsal part of the temporal stem, fibers in the depth of pars triangularis, and fibers belonging to the superior longitudinal fasciculus (SLF) (Figure 5). This pattern of result suggests a functional compartmentalization of the IFOF, with distinct sublayers at the subcortical level (stem of the bundle) as well as regarding their terminal parts. More specifically, a “ventral” IFOF sublayer may be implicated in visual semantic processing whereas a “dorsal” IFOF layer may be rather implicated in mentalizing with an additional contribution of the SLF, as previously suggested in lesion mapping studies (Herbet et al., 2014; Grosse Wiesmann et al., 2017).

This interpretation may be criticized on the ground that the subtraction analyses revealed only low to moderate differences between streamline distributions associated with semantic cognition vs. mentalizing and vice versa (max 25%). This was, however, expected for anatomical reasons. Indeed, a large proportion of stimulation sites was unmasked within the temporal stem. Given the width of this bottleneck structure [around 11 mm at the level of the limen insulae (Martino et al., 2010)] and the spatial resolution of the bipolar stimulator (5 mm), dissociating IFOF WM subpathways in this area is actually challenging. Besides, as reported by Wu et al. (2016), the fibers forming the different IFOF layers are relatively intermingled in the temporal stem, not organized into well-structured bundles. Consequently, the difference we found in the subtraction analyses were not so large because related to subcortical sites (and their associated streamlines) located outside the temporal stem, which were clearly less numerous. Note that in a recent study aimed at disentangling the motor-speech from the lexical semantic fiber pathways, comparable differences in streamline distribution was observed (a quite similar approach was used) (Corrivetti et al., 2019).

Remarkably, a high degree of anatomo-functional variations was identified across patients at both the cortical and subcortical level. More specifically, four patterns were observed: (i) a contribution to both categories of functions; (ii) a unique contribution to semantic cognition; (iii) a unique contribution to mentalizing; (iv) no contribution at all—knowing that mentalizing was over-represented compared with visual semantic cognition. Before elaborating further on this finding, two notes of caution are necessary. First, the fact that no response was identified for either mentalizing or semantics (or both), and most notably at the subcortical level, does not necessary mean that the IFOF was not implicated. Indeed, as patients were operated on according to the individual functional margins (i.e., the surgery procedure stopped when a functional response was repeatedly evoked during stimulation), some IFOF fibers were not stimulated, typically those running in the deeper component of the tract. Second, the pattern “no contribution at all” is quite artificial because uniquely appropriate when considering the cortical and subcortical levels, independently. Indeed, according to the inclusion criteria, patients took place in this study if at least one positive response was observed during the mapping. In other words, a few patients could show a functional response at the cortical level but not at the subcortical level and *vice versa*; this is not surprising because again all IFOF fibers were not stimulated and only the exposed cortical surface during surgery was stimulated. This implies, in the latter case, that some important cortical sites could have been missed because not accessible to the surgery procedure. With these limits in mind, our results nevertheless suggest an across-subject anatomo-functional variation that may be related to hemispheric dominance. This is especially reminiscent of a study by Catani et al. (2007) in which interhemispheric differences were found in the WM connections of the language network, from strongly left lateralized to bilateral symmetrical distribution. Consequently, a valuable extension of our study would be to assess to what extent semantic and mentalizing performances are related to left-right differences in network topology by combining fMRI and diffusion imaging in healthy participants. Besides, it is worth recalling that handedness may influence the interindividual variations we discussed above, as subcortical sites for mentalizing were less frequent in left-handed vs. right-handed patients.

Last, a few cortical and subcortical sites were associated with both types of functional responses. This result can be interpreted in two ways: either both functional networks were impaired by stimulation, meaning that the spatial resolution of stimulation was not enough to mark topological differences, or a more domain-general control or attention network was temporary disrupted. The latter explanation has to be prioritized, as the aspecific sites were observed uniquely at the cortical level in dlPFC, a region which is known to be involved in such networks (Chein and Schneider, 2005; Duncan, 2010). Besides, at the subcortical level where amodal sites were also observed, the IFOF is believed to support some kinds of amodal processing, especially in the context of semantic cognition (Moritz-Gasser et al., 2013).

The current study had some limitations. First, the positioning of anatomical sites relied on the use of anatomical landmarks identifiable on structural MRI and intraoperative photos. This procedure could lead to potential inaccuracies, but we showed an excellent inter-rater reliability in this mapping work – as in another recent work (Herbet et al., 2019). Second, a template-based approach was adopted to generate streamline tractograms from stimulation sites. Admittedly, the use of patient diffusion data would have been better. However, there are important difficulties to perform streamline tractography in subcortical structures impaired by lesional infiltrations. Third, low-grade gliomas are disproportionally located in paralimbic areas, implying that posterior structures were less stimulated. A larger set of posterior sites would have certainly help better dissociating the IFOF sublayers. Last, even if the IFOF is considered as a poorly compensable tract (Herbet et al., 2016), we cannot rule out that the possibility that neuroplastic compensations occurred.

In brief, the current study not only confirms that the network mediated by the IFOF is implied in both visual semantic cognition and mentalizing, but also suggests that the tract is dissociable into two functional strata according to a ventral (semantic) and dorsal (mentalizing) compartmentalization. Although this general finding needs to be replicated in healthy participants with other imaging modalities (e.g., coupling navigated transcranial magnetic stimulation with diffusion MRI to track white matter pathways from cortical sites), from a clinical standpoint, they are of central interest for the surgical planning of patients presenting with a tumor in the vicinity of this anatomical connectivity.

## Data Availability Statement

The original contributions presented in the study are included in the article/supplementary material, further inquiries can be directed to the corresponding author/s.

## Ethics Statement

Ethical review and approval was not required for the study on human participants in accordance with the local legislation and institutional requirements. The patients/participants provided their written informed consent to participate in this study.

## Author Contributions

AR, HD, and GH: conception and design of the study. AR, A-LL, HD, and GH: acquisition and analysis of data and drafting a significant portion of the manuscript and figures. JD and SN: analysis of data. SN, HD, and GH: critical review of the manuscript. All authors contributed to the article and approved the submitted version.

## Conflict of Interest

The authors declare that the research was conducted in the absence of any commercial or financial relationships that could be construed as a potential conflict of interest.

## Footnotes

1 http://www.fil.ion.ucl.ac.uk/spm/

2 http://marsbar.sourceforge.net

3 https://www.ibm.com

4 https://R-project.org/

5 http://dsi-studio.labsolver.org
